# Association Between the Lateral Wall Thickness of the Maxillary Sinus and the Dental Status: Cone Beam Computed Tomography Evaluation

**DOI:** 10.5812/iranjradiol.6675

**Published:** 2014-01-30

**Authors:** Saeedeh Khajehahmadi, Amin Rahpeyma, Seyed Hosein Hoseini Zarch

**Affiliations:** 1 Dental Research Center, Faculty of Dentistry, Mashhad University of Medical Sciences, Mashhad, Iran; 2 Oral and Maxillofacial Diseases Research Center, Faculty of Dentistry, Mashhad University of Medical Sciences, Mashhad, Iran

**Keywords:** Cone-Beam Computed Tomography, Maxillary Sinus, Mouth, Edentulous

## Abstract

**Background:**

Assessment of the lateral wall thickness of the maxillary sinus is very important in decision making for many surgical interventions. The association between the thickness of the lateral wall of the maxillary sinus and the dental status is not well identified.

**Objectives:**

To compare the thickness of the lateral wall of the maxillary sinus in individuals with and without teeth to determine if extraction of the teeth can lead to a significant reduction in the thickness of the maxillary sinus lateral wall or not.

**Patients and Methods:**

In a retrospective study on fifty patients with an edentulous space, the thickness of the lateral wall of the maxillary sinus,one centimeter above the sinus floor in the second premolar (P2), first molar (M1) and second molar (M2) areas was determined by cone beam computed tomography scans(CBCTs) and a digital ruler in Romexis F software (Planmeca Romexis 2.4.2.R) and it was compared with values measured in fifty dentated individuals. Three way analysis of variance was applied for comparison after confirmation of the normal distribution of data.

**Results:**

The mean of the wall thickness in each of these points was lower in patients with edentulous spaces; however it was not significant. There was no association between gender and the thickness of the lateral wall of the maxillary sinus, but location was associated with different thicknesses.

**Conclusions:**

The differences in the thickness based on the location and dental status necessitates assessment of the wall thickness of the maxillary sinus in addition to the current evaluation of bone thickness between the sinus floor and the edentulous crest before maxillary sinus surgery.

## 1. Background

Assessment of the thickness of the lateral wall of the maxillary sinus is very important in decision making for many surgical interventions such as Caldwell-Luc surgery, Lefort I osteotomy, open sinus lift, facial and jaw bone fracture fixation and mini-screw insertion in orthodontics as well as the diagnosis of chronic sinusitis (1-5). It is helpful in Caldwell-Luc surgery for producing a window to access the sinus cavity, in Lefort I osteotomy for exerting straight or stepped osteotomy, in internal fixation of maxillary fractures for selecting the appropriate length of titanium screws, and in open sinus lift surgery for estimating the approximate difficulty of the procedure (6). However, in spite of the importance of this issue, there are very few studies that have evaluated the anatomic features and more specifically, the thickness of the lateral wall of the maxillary sinus. It is assumed that extraction of the teeth might lead to a decrease in the thickness of the sinus wall.

## 2. Objectives

We designed a study to compare the thickness of the lateral wall of the maxillary sinus in individuals with and without teeth to determine if extraction of the teeth can lead to a significant reduction in the thickness of the maxillary sinus lateral wall or not. The number of years that had passed after teeth extraction was not considered in this study.

## 3. Patients and Methods

**Figure 1.**
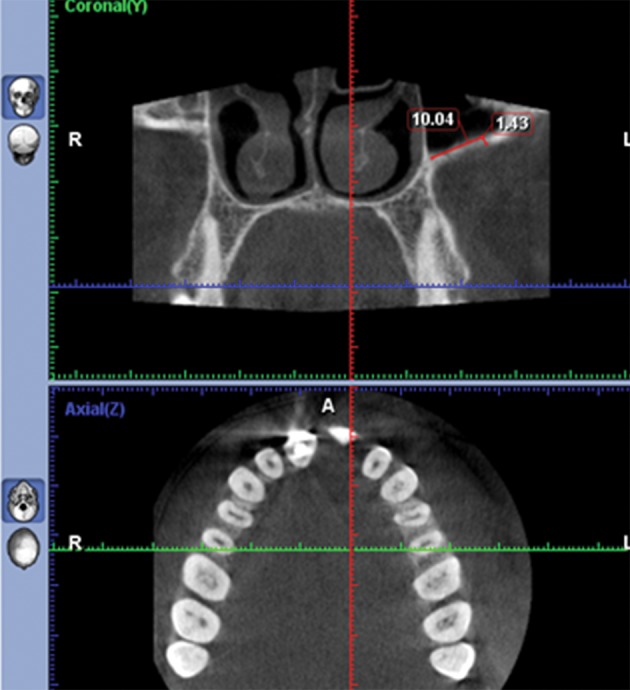
The thickness of the bone was measured in the second premolar (P2) 1 cm above the sinus floor.

**Figure 2.**
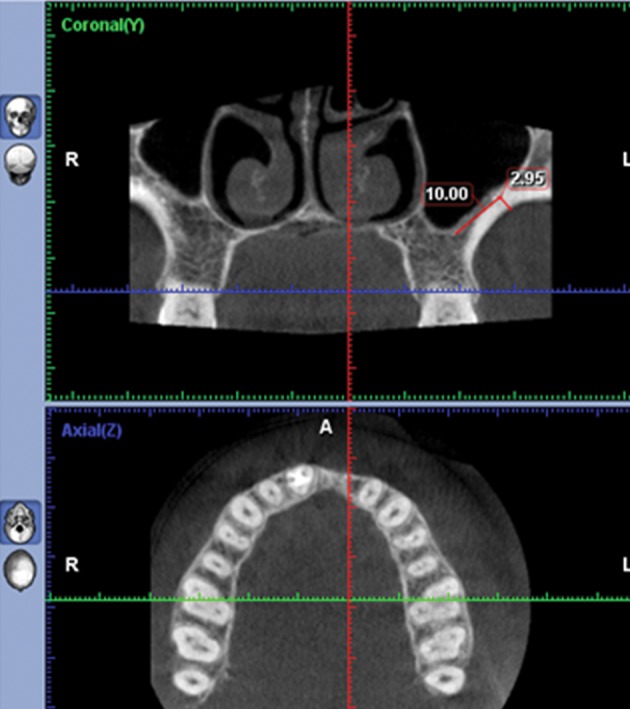
The thickness of the bone was measured in the first molar (M1) 1 cm above the sinus floor.

**Figure 3.**
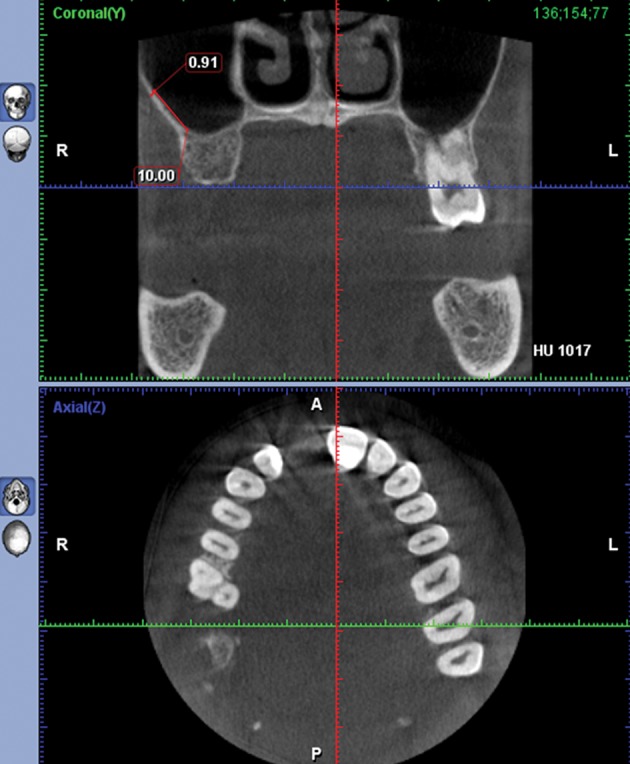
The thickness of the bone was measured in the second molar (M2) 1 cm above the sinus floor.

In this retrospective study, we assessed the thickness of the lateral wall of the maxillary sinus one centimeter above the sinus floor by cone beam computed tomography scan (CBCT) in fifty patients with edentulous spaces who were candidates for dental implant placement (the edentulous space group) and we compared the results with CBCTs of fifty maxillofacial trauma patients who had no maxillary bone and teeth problem (the dentate group). This area was chosen because the majority of surgical procedures that need bone removal to get access inside the maxillary sinus or osteotomy cuts and osteosynthesis devices are all involved with this location. The main inclusion criteria were the tooth absence and presence in the posterior maxilla in the coronal axis of CBCTs in the first and second groups, respectively. In addition, as the maxillary sinus fully develops in 15 year olds (7) and age may influence the thickness of the lateral sinus wall; cases were selected in the group of 40 to 60 year olds to better match the cases between the study groups. Furthermore, we tried to match them regarding gender by selecting 25 males and 25 females in each group (50 patients). Three hundred sites were measured; 150 sites in the dentate group and 150 in the edentulous group. In each group, half the measurements were done in males. In every gender, 25 sites were measured in the first premolar as well as the first and second molars. The exclusion criteria were positive history of maxillary fracture, acute or untreated chronic sinusitis or any other pathological lesion involving the maxillary sinus, including tumors, cysts, and previous regional surgery such as Cadwell-Luc. All the CBCTs were made by Promax 3D (Planmeca Co., Helsinki, Finland) at 0.16 mm pixel resolution, 8 kV, 8 mA and 12 seconds. Using 2-mm-thick reconstruction algorithms, the axial images were reconstructed into para-axial cross sections. Then, the thickness of the bone was measured in the second premolar (P2), first molar (M1) and second molar (M2) areas exactly one centimeter above the sinus floor. For this purpose, we used a digital ruler in Romexis F software (Planmeca Romexis 2.4.2.R) (Figure 1, 2 and 3). The sensitivity of this measurement was 0.01 mm. For confirmation of the data obtained from measurement by Romexis F software, re-measurement was performed for 20% of the cases. Intrarater reliability was high for both measurements of thickness (r=0.93). Data analysis revealed a normal distribution of the sample (by kolmogorovsmirnov test); therefore, multivariate analysis and repeated measures ANOVA wereusedfor statistical analysis.

## 4. Results

Multivariate analysis was used for comparison of lateral wall thickness of the maxillary sinus as the effects of gender and dental status were considered in the analysis. It showed that both of them and their interaction had no significant effect on the wall thickness (P>0.05)(Table 1). 

**Table 1.** Mean and Standard Deviation of the Lateral Wall Thickness of the Maxillary Sinus in Three Locations, According to Gender and Dental Status

Group	Tooth, mean±SD		
	P2	M1	M2
**Edentulous space**			
Male	1.47±0.26	2.88±0.56	0.89±0.16
Female	1.47±0.30	2.86±0.58	0.81±0.24
**Dentate**			
Male	1.56±0.31	3.03±0.53	0.90±0.21
Female	1.56±0.30	2.97±0.64	0.87±0.21
**P-Value**^**a**^	0.953	0.723	0.172
**P-Value**^**b**^	0.136	0.275	0.391

^a^ Genders

^b^ Dental status

**Figure 4.**
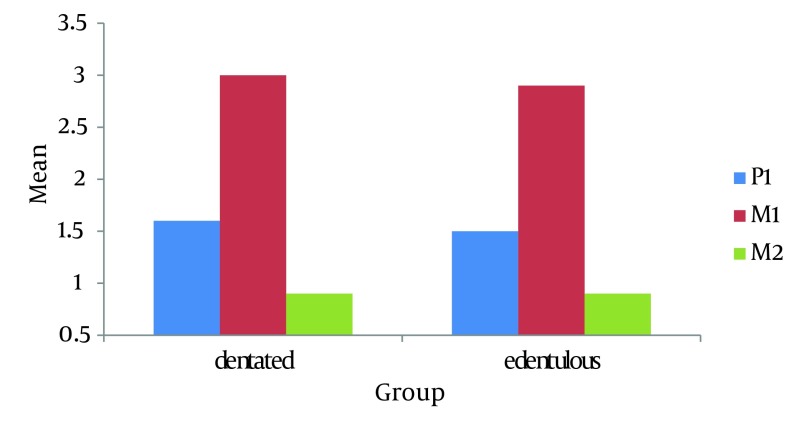
Mean of the bone thickness in the three locations (second premolar P2, first molar M1 and second molar M2)

For comparison between the three bony locations (second premolar, first molar and second molar), repeated measure ANOVA was used. There were significant differences between these three locations. Bonefferoni correction showed significant differences between all three pairwise locations (P<0.05). The thickest bone was thefirst molar region followed by the second premolar and finally the second molar (Figure 4). 

## 5. Discussion

While the anatomy of the maxillary sinus septa is well-identified (8-10), there are few studies about the topography of the lateral wall of the maxillary sinus to help surgeons who operate in this area. Arman et al. (11) evaluated 30 dry skulls for the thickness of the anterior wall of the maxillary sinus and revealed that there is no difference between the right and left side concerning the thickness of the wall. Our results showed that among the second premolar and first and second molar areas, the thickest wall was observed in the bone above the first molar and the least thickness was documented in the second molar. It seems that the increased thickness of the lateral wall of the maxillary sinus in the bone above the first molar is secondary to the presence of the buttress of the zygoma. This structure is a part of the maxillary bone that is attached to the zygomatic bone. One of the practical implications from the findings of this study is that application of 2-mm-thickness miniplates and 5-mm-length titanium screws can safely prevent insertion into the maxillary sinus cavity. Furthermore, according to the results of this study, because the mean thickness of the zygomatic buttress is 3 mm, using this part as a graft for the edentulous anterior maxillary bone would be insufficient in conditions that thick graft is needed (3). When there is an insufficient bone height in the maxillary posterior edentulous region, to insert dental implants with a sufficient length without perforating the maxillary sinus floor, open sinus lift surgery is indicated. In open sinus lift surgery, the increased thickness of the maxillary sinus wall is considered as a difficulty factor (6). Because higher thickness makes surgery harder and longer, knowing the thickness of the maxillary sinus wall would help the surgeon to select locations with a lower thickness to prevent surgical complications such as membrane perforation (6). In addition, piezosurgery is not recommended in the thick maxillary sinus wall as it is associated with an increased length of surgery (12). One important point especially in the Cadwell-Luc and open sinus lift surgery is that the maxillary sinus wall has a considerable vascular anastomosis (13) and nutritional canals with 2-3 mm diameter are observed in 7 percent of the individuals (14). It is more likely to find wider canals in the thicker wall (6); therefore, the bony window must be made with more consideration in thick sinus walls to prevent any unexpected bleeding during Cadwell-Luc and open sinus lift surgery.

The thickness of the maxillary sinushas previously showed to have association with the difficulty of sinus surgeries.The differences inthe thickness based on the location and dental status necessitates CBCT assessment of the wall thickness of the maxillary sinus in addition to the current evaluation of the bone thickness between the sinus floor and the edentulous crest or dental roots before sinus surgeries.
